# Timing and Patterns of Potentially Salvageable Recurrences Following Stereotactic Body Radiation Therapy for Clinically Localized Prostate Cancer Assessed by Preferential Amino Acid Uptake

**DOI:** 10.7759/cureus.77964

**Published:** 2025-01-25

**Authors:** Tim Kearney, Lauren Nagel, Matthew Bourne, Alan L Zwart, Deepak Kumar, Malika Danner, Simeng Suy, Michael Carrasquilla, Giuseppe Esposito, Sean Collins

**Affiliations:** 1 Radiation Oncology, MedStar Georgetown University Hospital, Washington, DC, USA; 2 Radiation Oncology, University of Florida, Gainesville, USA; 3 Radiology, MedStar Georgetown University Hospital, Washington, DC, USA; 4 Radiation Oncology, Julius L. Chambers Biomedical/Biotechnology Research Institute, North Carolina Central University, Durham, USA; 5 Radiation Oncology, Tampa General Hospital, Tampa, USA; 6 Radiation Oncology, Georgetown University, Washington, DC, USA; 7 Nuclear Medicine, MedStar Georgetown University Hospital, Washington, DC, USA

**Keywords:** cyberknife, external beam, prostate cancer, psma, sbrt

## Abstract

Purpose: 18F-fluciclovine is a radiolabeled amino acid analog that is preferentially taken up by prostate cancer cells. 18F-fluciclovine PET/CT scans are approved for the detection of biochemically recurrent prostate cancer. Stereotactic body radiation therapy (SBRT) is increasingly offered for the treatment of localized prostate cancer. Limited data exist on the patterns of failure following prostate SBRT. The impact of scan timing before or after meeting the Phoenix criteria is unknown. Here, we characterize 18F-fluciclovine-defined recurrences for patients with rising prostate-specific antigens (PSAs) following SBRT.

Methods: Between 2017 and 2022, 50 consecutive patients underwent an 18F-fluciclovine scan for suspected recurrence. All patients were treated on an institutional protocol with either SBRT (35-36.25 Gy) or SBRT boost (19.5 Gy) with intensity-modulated radiotherapy (IMRT). A total of 38% of the patients were high-risk, and 46% received androgen deprivation therapy (ADT) as part of their initial treatment. Patterns of failure were classified as PSA-only, local (prostate), lymph node (LN), bone, visceral, or combined. Patients were considered salvageable if all evidence of disease could be safely treated with local therapy (radiation, surgery, or interventional radiology (IR) ablation).

Results: The median time from treatment was 39 months, and the median pre-scan PSA was 2.8 ng/mL. The overall scan positivity rate in our cohort was 34/51 (67%). The most common sites for initial disease recurrence were the prostate (22%), pelvic and para-aortic lymph node basins (40%), and bone (6%). A total of 21/51 scans (41%) were performed prior to reaching the Phoenix definition (nadir + 2) at a median PSA of 1.14 ng/mL. Of these patients, 12 (57%) had evidence of disease recurrence, all of which were potentially salvageable local or LN recurrences. The remaining 30/51 (59%) scans were performed after meeting the Phoenix definition (median PSA = 5.65 ng/mL). Of these, 22/30 (73%) had disease recurrence and 82% were potentially salvageable.

Conclusions: The diagnosis and management of recurrence following prostate SBRT continues to evolve. Approximately 50% of patients in our cohort who had yet to meet the Phoenix definition had scan evidence of disease recurrence, all of which were potentially salvageable with additional local therapy. Additional research is needed to identify factors predictive of disease recurrence on 18F-fluciclovine scans prior to reaching the Phoenix definition when they may be most curable.

## Introduction

Stereotactic body radiation therapy (SBRT) is a safe and effective treatment option for patients with localized prostate cancer (PCa) [1,2]. SBRT delivers high biologically effective doses (BEDs) in five treatment sessions or less, with seven-year biochemical disease-free survival (bDFS) rates of 80-95% for low to intermediate disease [1] and four-year bDFS rates of 77-87% for high-risk disease [2]. Although SBRT achieves high early biochemical relapse-free survival rates, patients may experience biochemical failure many years after treatment [3-5].

Rises in prostate-specific antigen (PSA) are utilized for the early detection of recurrent disease and commonly occur years prior to clinical failure [6]. The Phoenix criteria define biochemical failure after radiation therapy as at least a 2 ng/mL rise in PSA above the nadir [7,8]. Studies on the kinetics of PSA after SBRT have shown that PSA rises following SBRT may reflect local and/or distant recurrence [5]. PSA monitoring cannot differentiate between local, locoregional, or systemic recurrence [9]. Salvage therapy is potentially curative following biochemical recurrence (BCR) after SBRT [9,10]. Strategies are needed to optimize the accurate early identification of salvageable failures.

New radiotracers such as prostate-specific membrane antigen-positron emission tomography/computed tomography (PSMA-PET/CT) and 18F-fluciclovine have higher sensitivity and specificity than conventional imaging for detecting new or recurrent prostate cancer [11]. PSMA-directed tracers bind to the PSMA receptor, which is a membrane-bound enzyme with higher expression in PCa tissue than in benign tissue. 18F-fluciclovine is a fluorinated synthetic levorotatory leucine (L-Leucine) analog that enters the cell via amino acid transporters ASCT-2 and LAT-1. PCa exhibits amino acid metabolism alterations (upregulation of amino acid transporters ASCT-2 and LAT-1) [12]. With lower uptake in the kidneys than other available PSMA tracers and thus minimal excretion through the genitourinary tract to obscure radiologic findings, 18F-fluciclovine is ideal for evaluating pelvic malignancy [13,14].

Fluciclovine significantly impacts patient management and outcomes [15,16]. Utilization of these scans has been shown to increase failure-free survival (FFS) compared with conventional imaging-guided radiotherapy planning [15]. It may be valuable in managing PCa patients showing early recurrence after SBRT. Limited data exist on the patterns of failure following prostate SBRT. The impact of scan timing before or after meeting the Phoenix Criteria is unknown. Others have suggested that promising biomarkers other than PSA, such as PSMA and fluciclovine, should be further evaluated to better identify those with salvageable PCa at an earlier time after SBRT [12,17,18]. Here, we characterize 18F-fluciclovine-defined recurrences for patients with rising PSAs following SBRT.

This article was previously presented as an abstract at the 2023 annual ASTRO meeting on October 3, 2023.

## Materials and methods

This retrospective study included 50 patients who underwent 18F-fluciclovine PET/CT scans for suspected recurrence between 2017 and 2022 after definitive SBRT for localized PCa at MedStar Georgetown University Hospital. All patients were treated on an institutional protocol with either SBRT 35-36.25 Gy/5 fractions or SBRT 19.5 Gy/3 fractions using the CyberKnife robotic radiosurgical system (Accuray Inc., Sunnyvale, CA, USA), with supplemental intensity-modulated radiotherapy (IMRT) to 45-50.4 Gy. SBRT and IMRT treatment methods have been detailed previously [19,20]. Patients were followed up with clinical evaluations and PSA levels. Institutional IRB approval was obtained for a retrospective review of data prospectively collected in our institutional database (IRB 09-510).

Patients who experienced a rise in serum PSA level after radiotherapy during the follow-up period underwent imaging for restaging at the discretion of the treating physician (SPC) using 18F-fluciclovine PET/CT. If more than one scan was performed for a patient in the follow-up period, only the findings of the first scan post-biochemical failure were included for analysis.

Recurrent lesions on the 18F-fluciclovine PET/CT scan were defined using a combination of morphology and radiotracer uptake. Focally increased radiotracer uptake, more than the background or visible morphological lesions, was considered positive for disease recurrence [13]. A local and/or distant disease (lymph node, bone, or viscera) recurrence was denoted if the lesion was greater than 1 cm in maximal dimension and exhibiting activity higher than the mean bone marrow activity or if the lesion was sub-centimeter and exhibiting activity higher than the mean blood pool activity and visually approaching that of the marrow [13]. Up to five sites of recurrence apart from the prostate gland were classified as oligometastases [21,22]. Patients were considered salvageable if they met these criteria and if all evidence of disease could be safely treated with local therapy (radiation, surgery, or interventional radiology (IR) ablation).

We noted several patterns of failure. Biochemical failure was classified as PSA-only if no malignancy was seen on the scan. Local failure was classified as occurring only in the prostate. Lymph node (LN) failure was classified as positive pre-sacral, perirectal, common iliac, internal, external, or obturator nodes. We also noted bone failure, visceral failure, and combined.

## Results

Fifty patients who underwent 18F-Fluciclovine PET/CT scans for suspected recurrence between 2017 and 2022 after definitive SBRT for localized PCa at MedStar Georgetown University Hospital were eligible for inclusion in this retrospective review. A total of 51 scans on 50 patients were performed. An additional scan (PSA of 4.8 ng/mL) was performed on one patient, which showed evidence of disease with a local-only recurrence after an initial negative scan (PSA of 2.8 ng/mL). Patient characteristics are shown in Table 1.

**Table 1:** Patient characteristics SBRT: stereostatic body radiation therapy; IMRT: intensity-modulated radiation therapy

Patient Characteristics	N = 50 (%)
Gleason score	
3+3	17 (34)
3+4	7 (14)
4+3	5 (10)
4+4	13 (26)
4+5	7 (14)
5+5	1 (2)
T category	
T1c	19 (38)
T2a	8 (16)
T2b	13 (26)
T2c	7 (14)
T3	3 (6)
N category	
Nx	30 (60)
N0	18 (36)
N1	2 (4)
Risk stratification	
Low risk	1 (2)
Favorable intermediate risk	8 (16)
Unfavorable intermediate risk	20 (40)
High-risk	11 (22)
Very high-risk	8 (16)
Regional	2 (4)
Androgen deprivation therapy (ADT)	23 (46)
Length of ADT	
Short-term	17 (74)
Long-term	6 (26)
Treatment delivery	
SBRT alone	34 (68)
IMRT + SBRT boost	16 (32)
Nodes treated	6 (12)

In total, 38% (19/50) of the patients were high or very high-risk, and 46% (23/50) received androgen deprivation therapy (ADT) as part of their initial treatment. The median pretreatment PSA was 11.4 ng/mL. Patients had a median PSA of 2.8 ng/mL and a median PSA doubling time of 10 months at the time of the 18F-fluciclovine PET/CT scan, with a median time from the treatment of 39 months. The median time to nadir (no ADT) was 18 months, and the median PSA nadir was 0.3 ng/mL. Table 2 presents the results of the 18F-fluciclovine scans.

**Table 2:** Results of the 18F-fluciclovine scans PSA: prostate-specific antigen

Parameters	Number of Positive Scans (N=51)	Percentage (%)
Overall scan positivity rate	34	66.7
Pelvic/para-aortic lymph node recurrence	20	39.2
Prostate recurrence	11	21.6
Bone recurrence	3	5.9
Lymph node-only recurrence	19	95
Lymph node + visceral recurrence	1	5
Median number of lymph nodes involved	2 (range: 1–12)	-
Oligometastatic recurrences	20	39.2
Confirmed recurrences with additional imaging	44	86.3
Benign PSA bounce	1	2

The overall scan positivity rate was 34/51 (67%). The most common sites for initial disease recurrence were pelvic and/or para-aortic lymph node basins (40%), prostate (22%), and bone (6%). Among the recurrences to the lymph nodes, 19 (95%) were confined to the lymph nodes only, and one (5%) was found to be both visceral and to the presacral lymph nodes. The median number of lymph nodes involved was two and ranged from one to 12. A total of 20 (39%) recurrences were oligometastatic, with 19 cases involving the lymph nodes and one involving the bone. Out of 51 scans, 44 (86%) confirmed recurrences with additional imaging (MRI or PSMA-PET/CT), biopsy, or a nadir +2. One scan was performed on a patient who was later found to have a benign PSA bounce [23]. Of the 51 scans, 21 (41%) were performed before reaching the Phoenix definition (nadir + 2) at a median PSA of 1.14 ng/mL (Table 3).

**Table 3:** Results of the 18F-fluciclovine scans relative to the Phoenix definition

Scan Timing	N (%)	Median PSA (ng/mL)	High/Very High-Risk Patients (%)	Patients With Disease Recurrence (%)	Median PSA at Recurrence (ng/mL)	Salvageable Recurrences (%)
Before Phoenix definition	21 (41)	1.14	48	12/21 (57)	1.05	57
After Phoenix definition	30 (59)	5.56	33	22/30 (73)	7.05	82

Approximately 33% of patients scanned after meeting the Phoenix criteria were high-risk or very high-risk. Of these patients, 12/21 (57%) had evidence of disease recurrence (median PSA = 1.05 ng/mL). All were potentially salvageable local (19%) or LN (38%) recurrences. A total of 48% of patients scanned before reaching the Phoenix criteria were high-risk or very high-risk. The remaining 30/51 (59%) scans were performed after meeting the Phoenix definition (median PSA = 5.56 ng/mL). Of these, 22/30 (73%) had disease recurrence (median PSA = 7.05 ng/mL), and 18 (82%) were potentially salvageable.

## Discussion

Our institutional experience adds to the growing body of evidence supporting the value of 18F-fluciclovine PET/CT scans in identifying potentially salvageable recurrences following definitive radiation therapy for prostate cancer [24]. To our knowledge, this is the first study specifically looking at 18F-fluciclovine PET/CT sensitivity following prostate SBRT. 18F-fluciclovine PET/CT identified at least one recurrent lesion in 67% of our patients at a median PSA of 2.8 ng/mL. This is consistent with rates reported by other studies for alternative patient populations (26-79%: Table 4) [24]. Similar to other studies, our detection rate increased with increasing PSA values.

Interestingly, our study showed a relatively low rate of local recurrences [5] and a high rate of nodal oligorecurrences [25]. This may be at least partially due to the high BEDs delivered via prostate SBRT. All recurrences are discussed in a multidisciplinary setting. At our institution, focal recurrences are usually addressed with a CyberKnife SBRT protocol, treating up to 34Gy/5 fractions to the gross lesion, assuming dose constraints can be met, with concurrent ADT. Nodal recurrences are usually addressed with pelvic lymph node IMRT and concomitant ADT. Focal re-irradiation of post-RT prostatic recurrences is an area of active clinical investigation [26]. Unfortunately, fluciclovine scans have low specificity for disease in irradiated prostate [27]. This could be due to uptake in benign prostate tissue and prostatic inflammation. The authors believe that F18-labelled PSMA agents, with their enhanced PET scan resolution, are preferred for this indication. Due to the low renal excretion and high specificity for involved nodes, fluciclovine scans may be the ideal imaging agent to identify and treat nodal oligorecurrences [28]. This is important, as two prevailing hypotheses exist: one views nodal metastases as a precursor to widespread disease, while the other considers them a distinct metastatic lineage. Despite this, the clinical benefit of treating nodal recurrences through metastasis-directed therapy (MDT), such as SBRT, has gained traction, particularly given its potential to prolong progression-free survival and delay systemic therapy initiation. This can be accomplished through the utilization of involved-field SBRT to PSMA-directed targets [25].

Early detection of recurrences is potentially clinically important. Our overall detection rate increased to 73% in patients who had met the Phoenix criteria for biochemical failure. However, the percentage of patients with potentially salvageable recurrences was higher in those with rising PSA but were yet to meet the Phoenix criteria (100% versus 82%). Similarly, multi-variable analysis in the EMPIRE study showed that fluciclovine scans improved FFS in patients with rising PSAs who had not yet reached the Phoenix criteria prior to salvage RT. 18F-fluciclovine PET/CT improved RT targeting by enabling precise field adjustments. This translated into significant gains in event-free survival (EFS), especially among patients with PSA < 2 ng/mL, highlighting its utility for early intervention [29,30].

PSMA and fluciclovine transports may have variable expression in prostate cancer subtypes. A recent retrospective review looking at the expression of genes encoding targets for 18F-fluciclovine and 68Ga-PSMA-11 in ~18,000 prostate cancer specimens showed that PSMA expression is positively associated with unfavorable features, whereas higher expression of LAT3 and ASCT2 (which encode for the targets of the radiolabeled tracers) was associated with improved clinical outcomes [25]. PSMA expression predicted an increased risk of BCR, while LAT3, LAT4, and ASCT2 were linked to reduced BCR risk. We thus hypothesize that recurrences with high fluciclovine transporter expression could be more responsive to local salvage approaches such as SBRT.

This study has several limitations. First, the suspected sites of recurrence were not uniformly confirmed by biopsy. However, the scans in this study were interpreted by a highly experienced reader (GE), and prior research has suggested that 18F-fluciclovine PET/CT is highly specific, limiting the potential benefit of biopsy confirmation [31]. Second, our definition of salvageable recurrence may not be accepted by all readers, lessening the generalizability of our work. Finally, 18F-fluciclovine PET/CT scans may have less diagnostic accuracy than the currently available PSMA scan [32,33].

## Conclusions

The diagnosis and management of recurrence following prostate SBRT continue to evolve. Approximately 50% of patients in our cohort who had yet to meet the Phoenix definition had scan evidence of disease recurrence, all of which were potentially salvageable with additional local therapy. Additional research is needed to identify factors predictive of disease recurrence on 18F-fluciclovine scans prior to reaching the Phoenix definition when they may be most curable.
